# A Video App for Screening Osgood-Schlatter Disease Using Soccer Instep Kicking Motion Analysis

**DOI:** 10.7759/cureus.63112

**Published:** 2024-06-25

**Authors:** Tatsuo Fukuoka, Fumiya Ikubo, Kentaro Ono, Naoya Iwamoto, Yukinori Misaki, Yuto Kashihara, Hitoshi Yamada, Konosuke Yamaguchi, Tomohiko Hirose, Masakazu Ishikawa

**Affiliations:** 1 Department of Orthopaedic Surgery, Kagawa Saiseikai Hospital, Takamatsu, JPN; 2 Department of Rehabilitation Medicine, Faculty of Medicine, Kagawa University, Takamatsu, JPN; 3 Department of Orthopaedic Surgery, Kagawa Prefectural Shirotori Hosiptal, Higashikagawa, JPN; 4 Department of Electronic Systems Engineering, National Institute of Technology, Kagawa College, Mitoyo, JPN; 5 Department of Electronic Systems Engineering, D-yorozu Co. Ltd., Mitoyo, JPN; 6 Deapartment of Orthopaedic Surgery, Faculty of Medicine, Kagawa University, Takamatsu, JPN; 7 Department of Orthopaedic Surgery, Hirose Hospital, Takamatsu, JPN; 8 Department of Orthopaedic Surgery, Faculty of Medicine, Kagawa University, Takamatsu, JPN

**Keywords:** instep kick, knee flexion angle, soccer, two-dimensional motion analysis, osgood–schlatter disease

## Abstract

Background: Osgood-Schlatter disease (OSD) is a type of osteochondrosis and traction apophysitis that results from repeated contractions of the quadriceps femoris muscle on the tibial tuberosity. Its prevention, early diagnosis, and treatment are crucial because it causes chronic knee pain and surgical approaches are required if left untreated. Three-dimensional motion analysis is a useful approach for elucidating the pathological factors of OSD; however, it requires advanced cameras, sophisticated facilities, and expensive software. Conversely, the advent of technology has provided affordable video recording devices, and smartphone/tablet-based applications have enabled two-dimensional (2D) motion analysis. This emerging tool and artificial intelligence technology were used to analyze the pivot leg from videos recorded on a tablet device during the instep kicks of adolescent soccer players. Therefore, in this study, we aimed to determine whether the pathological factors for OSD occurring in the pivot foot can be identified through a simple 2D motion analysis using a tablet device.

Methods: In total, 94 knees of 47 soccer players (aged 14.1±0.8 years, all male) who belong to a single soccer club were evaluated. OSD was diagnosed using ultrasonography and physical examination (a positive bone fragment on ultrasonography or tenderness at the tibial tuberosity). Lower limb muscle tightness was evaluated using the finger-floor distance, straight leg raising test, heel-buttock distance, Thomas test, and ankle range of motion using a goniometer. We then performed motion analysis, and the instep kicking motion was recorded using a video camera on a tablet device. The joint angles of the hip, knee, and ankle were measured using a real-time human-pose detection system. Data were compared between the OSD and non-OSD groups.

Results: Overall, six of the 47 players (12.8%) were diagnosed with OSD. No correlation was found between lower limb tightness and the occurrence of OSD in all indices. However, the 2D motion analysis revealed that the knee flexion angle at the time of plantar placement during the instep kick movement was significantly larger in the OSD group than in the non-OSD group (OSD group: 42.0±7.2˚, non-OSD group: 33.5±6.6˚, *p=0.013).

Conclusion: A video motion analysis revealed that the knee flexion angle during the instep kicking motion was significantly greater in athletes with OSD of the supporting foot.

## Introduction

Osgood-Schlatter disease (OSD) is a typical disorder affecting adolescent soccer players. Bony repair is feasible in over 90% of patients with early and appropriate treatment [1]. However, if proper diagnosis and treatment are delayed, avulsed bone fragments can cause chronic inflammation and pain, leading to a loss of support activity [2]. Therefore, early diagnosis, treatment, and understanding of OSD pathophysiology are critical.

However, for the early diagnosis and prevention of OSD, medical checks for young soccer players have been performed [3,4], which require significant human resources and time. Therefore, it is necessary to create a device that can perform convenient medical checks. Previous studies have used three-dimensional (3D) motion analysis to determine the risk factors for OSD [5,6]. Takei et al. reported a significant difference in the pivot point of kicks between athletes with and without OSD and concluded that the OSD group had significantly faster knee joint extension speeds of the supporting leg at the moment of impact with the ball than the non-OSD group [5]. The role of the supporting leg during the kicking motion is to lift the trunk upward by extending the knee joint of the supporting leg at the time of ball impact so that each velocity image of the extension of the knee joint of the supporting leg matches the speed of the kicking leg and ball. They are also involved in the process. Therefore, there are reports that the quadriceps of the supporting leg become overactive during kicking rather than the kicking leg [7-9].

Thus, 3D motion capture, especially the kicking motion, is a reasonable approach to characterize and reveal risk factors in each athlete; however, it is not feasible for frequent medical checks and use in many candidates due to the specificity of its devices and cost. Therefore, we addressed this issue by focusing on two-dimensional (2D) video motion analysis using a tablet device. The 2D video analysis is an emerging tool for the kinematic assessment and observational measurements of various sports activities [10]. In addition, the accuracy of identifying key body landmarks from video imaging (2D poses) without specific markers based on artificial intelligence (AI) models using deep neural network technology has been well established and used to evaluate posture behavior in actual work environments [11-13].

Therefore, in this study, we aimed to investigate whether 2D video motion analysis, captured with a tablet device using AI technology, will describe specific features of the instep kicking motion in adolescent athletes with OSD compared with physical information.

## Materials and methods

This study was approved by the Ethics Committee of the Faculty of Medicine, Kagawa University Hospital and was conducted following the Declaration of Helsinki (approval number: 2023-156). All participants and their parents provided written informed consent before participating in the study. 

Participants

Overall, 47 soccer players from a single local soccer club team participated in this study. All players were junior high school students in their first and second years (all male; age, 14.1±0.8 years; height, 164.1±7.5 cm; body weight, 53.8±7.6 kg; body mass index, 19.9±2.1 kg/m^2^). We included all players in the team's U13/U14 categories and excluded the players who had any surgery on the lower limb. Their soccer experience was 7.1 ±2.0 years (age at started playing soccer, 7.0 ±1.9years) and they were all in good health without any chronic diseases. Table 1 shows information on height increase in the last one year, sleeping hours, and medical history that was collected during interviews before the physical examination. 

**Table 1:** Participant information Values represent mean ± standard deviation.

Number of participants	n = 47
Age (years)	14.1±0.8
Height (cm)	164.1±7.5
Body weight (kg)	53.8±7.6
Body mass index (kg/m^2^)	19.9±2.1
Soccer experience (years)	7.1±2.0
Age at started playing soccer (years)	7.0±1.9
Sleeping hours (hours)	7.5±0.8
Height increase last one year (cm/year)	5.0±2.9

Muscle tightness testing

Muscle tightness testing was performed using the following criteria: 1) finger-floor distance as paraspinal muscle tightness (FFD, cm); 2) distance between the knee fossa and floor as iliopsoas muscle tightness (Thomas test, °); 3) straight leg raising test as tightness of the thigh flexor muscle (SLR, °); 4) heel-buttock distance as tightness of the quadriceps femoris muscle (HBD, cm); and 5) active ankle dorsiflexion range of motion as triceps surae tightness (ROM of ankle,˚). All measurements were repeated twice by a single skilled physical therapist who demonstrated excellent intrarater reliability for muscle tightness measures. 

Physical examinations and ultrasonography of the tibial tubercle

**Figure 1:**
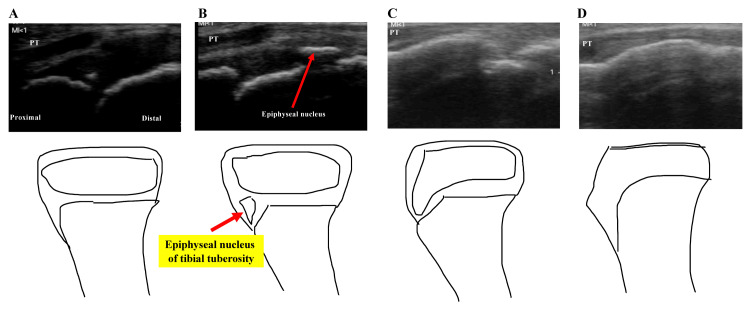
The bone growth stage of the tibial tuberosity Ultrasonographic morphological characteristics of the tibial tuberosity in sagittal view according to the Ehrenborg and Largergen classification. (A) Cartilaginous stage, (B) apophyseal stage, (C) epiphyseal stage, and (D) bony stage. PT, patella tendon. Source: [14]

Athletes who had tenderness of the tibial tuberosity were diagnosed with OSD. Ultrasound examination was performed to obtain a long-axis view of the tibial tuberosity (Pocket Echo Miruco, Sigmax, Japan). The bone growth stage of the tibial tuberosity was assessed based on an ultrasound using the Ehrenborg classification: cartilaginous, apophyseal, epiphyseal, and bony stages (Figure 1) [14].

Two-dimensional video kicking motion analysis 

**Figure 2:**
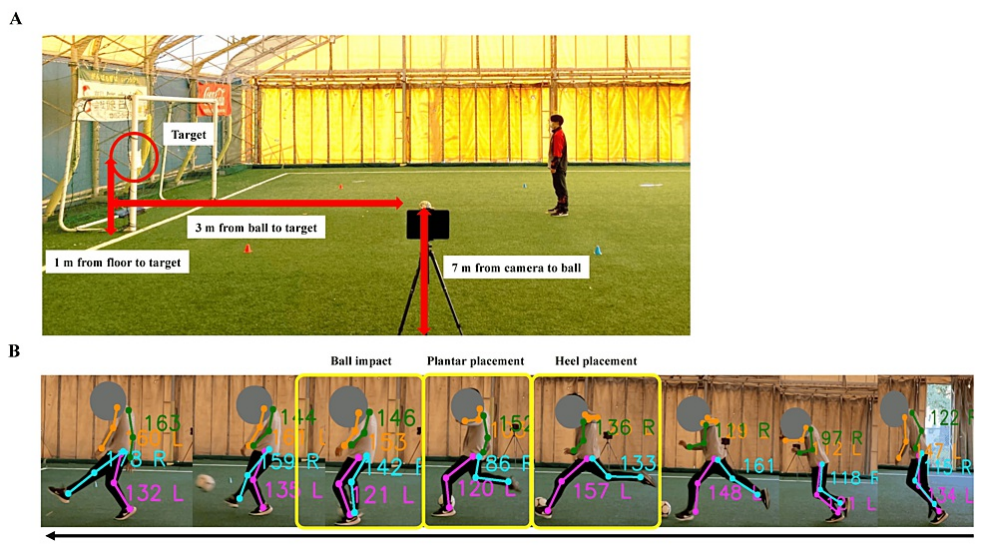
Two-dimensional video analysis of the motions in instep kicking (A) Setting of the ball, goal, and tablet device for instep kicking. (B) Motion sequences of instep kicking.

After physical examination and ultrasonography, instep kicking sequences were filmed using a video camera equipped with a tablet device, and the images were processed at 30 frames per second (iPad, Apple, USA). For the instep kick, the ball was placed 3 m from the goal on the indoor soccer field. The run-up distance for the kick was set at 3 m from the ball. A target was set at a height of 1 m from the floor at the center of the goal, and the participants were instructed to perform an instep kick with maximal effort toward the target. The tablet device was placed on the pivot foot side and the video recording was performed (Figure 2A). We used the captured video and investigated the flexion angle of the knee joint of the pivot leg at the following three points: 1) when the heel was placed just before the ball impact (heel placement), 2) when the sole was placed just before the ball impact (plantar placement), and 3) during the ball impact (ball impact). Video recordings were analyzed, and the ROM of the joints was measured using an AI-based human pose detection model, YOLOv8x Pose, which was pre-trained with the COCO-pose dataset [15]. The measurement was repeated thrice, and the average values were used for the analysis (Figure 2B). 

Statistical analysis

All statistical analyses were performed using GraphPad Prism software (GraphPad Software, MA, USA). Basic statistics of the measurement items are shown as mean value ± standard deviation. The Mann-Whitney U test was used for testing. The significance level was set at p < 0.05.

## Results

Overall, six of the 47 players (12.8%) were diagnosed with OSD. Four of the six players were diagnosed in both knees. In other words, 10 out of 94 knees of 47 players tested positive. Regarding the developmental stages of the tibial tubercle of a total of 94 knees, the epiphyseal stage was observed in seven knees and three knees demonstrated the bony stage in the OSD group. In the non-OSD group, three, 58, and 23 knees were in the apophyseal, epiphyseal stage, and bony stages, respectively (Table 2).

**Table 2:** Developmental stages of the tibial tubercle from ultrasound information Values represent means ± standard deviation. OSD, Osgood-Schlatter disease.

	OSD group (n = 10 Knees)	Non-OSD group (n = 84 Knees)
Cartilaginous stage	0	0
Apophyseal stage	0	3
Epiphyseal stage	7	58
Bony stage	3	23

The two groups showed no significant differences in height increase over one year, sleeping hours, or age at which the children started playing soccer. In addition, no significant differences were observed in any of the lower muscle tightness tests between the OSD and non-OSD groups. 

**Figure 3:**
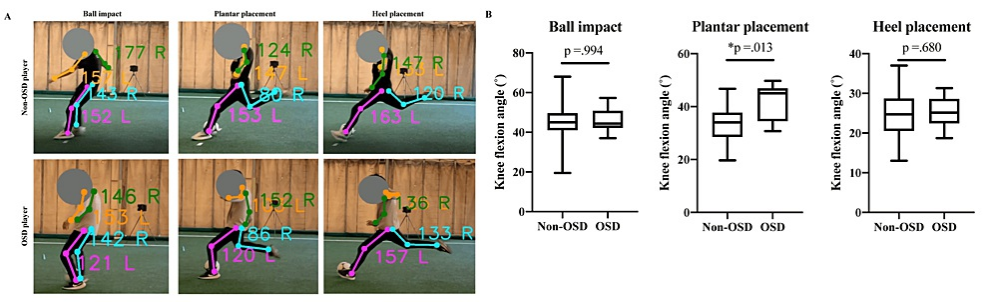
The difference in the knee flexion angle in instep kicking between OSD and non-OSD groups (A) Representative images of instep kicking of OSD and non-OSD players. (B) The difference in the knee flexion angle in the three points. There is a significant difference only in the time of plantar placement. *p<0.05 OSD, Osgood-Schlatter disease.

Regarding the pivot knee during instep kick motions, athletes with OSD demonstrated a significantly larger knee flexion angle at the time of plantar placement during the stepping motion just before ball impact (heel placement: OSD group: 25.3 ±4.3˚, non-OSD group: 24.3 ±5.4˚, p=0.680, plantar placement: OSD group: 42.0 ±7.2˚, non-OSD group: 33.5 ±6.6˚, *p=0.013, ball impact: OSD group: 46.0 ±6.7˚, non-OSD group: 45.5 ±9.8˚, p=0.994). However, there was no significant difference in the knee flexion angle at the time of heel placement and ball impact (heel placement: OSD group: 25.3 ±4.3˚, non-OSD group: 24.3 ±5.4˚, p=0.680, ball impact: OSD group: 46.0 ±6.7˚, non-OSD group: 45.5 ±9.8˚, p=0.994) (Figures 3A, 3B).

The flexibility of the lower limbs, as assessed by the indices, did not directly affect the knee flexion angle during the specific motion of an instep kick (Table 3).

**Table 3:** Comparison of parameters between OSD and non-OSD groups Values represent means ± standard deviation. OSD, Osgood-Schlatter disease; FFD, finger-floor distance; SLR, straight leg raising test; HHD, hip-heel distance; ROM, range of motion; 2D, two-dimensional.

		OSD group (n=6)	Non-OSD group (n=41)	p-value
Age (y)		15.0	15.0	0.25
Height (cm)		166.5±8.1	163.8±58.1	0.46
Height increase per year (cm)		4.9±3.3	5.7±2.7	0.45
Body weight (kg)		55.3±5.1	53.5±7.8	0.53
FFD (cm)		3.1±6.0	1.7±3.5	0.56
Thomas test (cm)	Dominant leg	4.7±1.1	4.3±1.4	0.39
	Pivot leg	5.1±1.7	4.3±1.4	0.15
SLR (˚)	Dominant leg	75.8±6.1	75.0±9.0	0.90
	Pivot leg	78.5±6.4	73.3±8.6	0.13
HBD (cm)	Dominant leg	15.6±3.6	13.7±3.5	0.29
	Pivot leg	16.2±4.8	14.2±3.6	0.18
ROM of ankle (˚)	Dominant leg	12.5±2.5	11.7±5.3	0.60
	Pivot leg	14.2±3.4	11.9±5.2	0.23

## Discussion

We investigated the characteristics of lower limb muscle tightness and instep kick movements in adolescent soccer players who developed OSD based on physical checks and 2D video analysis using a tablet device (iPad) and open-source software [15]. Muscle tightness of the lower limb evaluated with five muscle tests suggests that the flexibility of the lower limb was not associated with OSD. The 2D motion analysis can describe specific features of the instep kicking motion of the pivot leg. 

OSD has been thought to be due to excessive traction force applied to the quadriceps muscles during the apophyseal and epiphyseal stages when the tibial tuberosity is mechanically weak; however, the tightness of the quadriceps muscles is significant in patients with OSD [1,2,16]. Therefore, stretching of the quadriceps femoris muscle is recommended as a treatment strategy, as there are many reports of an increase in the size of the quadriceps muscle [17]. OSD has also been reported to occur more often in the pivoting leg than in the kicking leg [18]. The role of the pivot leg during the kicking motion is to lift the trunk upward by extending the knee joint of the pivot leg at the time of ball impact so that each velocity image of the extension of the pivot leg knee joint matches the speed of the kicking leg and ball. In the past, it has been reported that the pivot legs are involved. Therefore, there are reports that the quadriceps of the pivot leg become overactive during kicking rather than the kicking leg [7-9].

Takei et al. investigated the relationship between OSD and kicking motion using 3D motion capture. Takei et al. reported that in the OSD group, the longitudinal center of gravity shift and pelvic rotation during the preparatory period before ball impact were smaller, and the speed at which the knee joint of the pivot leg extended at the moment of ball impact was significantly faster than that in the non-OSD group [5]. Based on this report, it is suggested that we can detect athletes who are prone to OSD at an early stage and provide early guidance for intervention by observing the kicking motion during medical examinations. The 3D motion analysis is a useful approach for elucidating the pathological causes of OSD. However, it requires sophisticated equipment with advanced cameras and expensive software. Consequently, it is difficult to put this into practical use as a tool for early diagnosis and prevention in daily sports settings. In recent years, a highly versatile motion analysis method using an inexpensive video camera system and free software has recently been introduced, and its measurement accuracy has improved [10,19]. Therefore, we investigated whether the pathological factors for OSD that occur in the pivot foot can be identified through a simple 2D motion analysis using a tablet device and free software. The 2D motion analysis used in this study is a feasible approach. The kicking motion is captured within 3 min, which could be an advantage for applications in sports settings and home care. 

In our study, the angle of knee flexion at the moment of plantar placement during the stepping motion just before ball impact was significantly greater in athletes with OSD of the pivot leg during the instep kick motion. The OSD group had a deeper knee joint depression angle when stepping than the non-OSD group, but no difference was observed in the flexion angle at the time of ball impact. It is believed that force is required to greatly extend the knee joint. Consequently, the quadriceps femoris may have been overloaded. Using 2D motion analysis, we found significant differences in the instep kick motion between the OSD and non-OSD groups. Considering that 3D analysis requires the installation of multiple landmarks and high-performance cameras [5,20], being able to identify the pathological factors of OSD with simple preparation in a short time is extremely useful. 

However, there was no significant correlation between the flexion angle of the pivot knee and muscle tightness evaluation based on five muscle tests of the lower limb. Therefore, we could conclude on how these results relate to physical characteristics. In the present study, we were able to simply recognize some of the characteristics of the kicking motion in the OSD group. By expanding the scope of analysis to include more targets in the future, it might be feasible to detect and prevent OSD at an early stage by specifically analyzing the pivot foot during the kicking motion. This approach could provide valuable insights into the biomechanics of the kicking motion associated with OSD and help develop preventive measures or interventions.

This study has some limitations. First, owing to its cross-sectional nature, it is unclear whether these differences contribute to the occurrence of OSD or were a result of it. Therefore, longitudinal studies are warranted to elucidate the causal relationships between the variables studied and OSD development. Second, the limited number of participants may have affected the generalizability of the findings. Increasing the number of targets in future studies could provide a more comprehensive understanding of the factors influencing OSD. Third, differences in kicking skills among the participants may have influenced the results of the study. Future research could benefit from controlling for these differences or stratifying participants based on their skill levels to better isolate the effects of the variables under investigation. Finally, verifying the reliability of the joint range of motion measurements in the software used is also crucial. Ensuring the accuracy and consistency of the data collected from the software will enhance the validity of the findings or conclusions drawn from the analysis. This verification process helps to maintain the integrity of the research and ensures that the results are robust and dependable for further interpretation and application. Overall, although the current study provides valuable insights into the relationship between lower limb flexibility and OSD, these limitations highlight the need for further research to better understand the complexities of this relationship.

## Conclusions

A video motion analysis revealed that the knee flexion angle during the instep kicking motion was significantly greater in athletes with OSD of the supporting foot. This approach has the potential to determine the pathological factors of OSD more easily and cheaply than 3D motion analysis.
